# Right-to-Left Shunt via a Patent Foramen Ovale Triggered by Constrictive Pericarditis With Right Heart Compression: A Case Report

**DOI:** 10.7759/cureus.98123

**Published:** 2025-11-29

**Authors:** Takeshi Yamashita, Hanako Yoshihara Kurihara, Tamami Watanabe, Kenichi Sugisaki, Takahiko Fukuchi

**Affiliations:** 1 Division of General Medicine, Department of Comprehensive Medicine, Jichi Medical University Saitama Medical Center, Saitama, JPN; 2 Division of Radiology, Department of Comprehensive Medicine, Jichi Medical University Saitama Medical Center, Saitama, JPN

**Keywords:** constrictive pericarditis, hypoxemia, paradoxical cerebral embolism, patent foramen ovale (pfo), right-to-left cardiac shunt

## Abstract

We report a rare case of a right-to-left shunt through a patent foramen ovale (PFO) triggered by constrictive pericarditis secondary to an organized pericardial hematoma. A 72-year-old woman presented with thoracic back pain and subsequently developed persistent hypoxemia following idiopathic pericarditis. Despite appropriate anti-inflammatory and antimicrobial therapy, she experienced a cerebral embolism involving the right middle cerebral artery territory on hospital day 50. Imaging studies revealed a pericardial effusion compressing the right heart. Intraoperative transesophageal echocardiography confirmed a right-to-left shunt through a PFO. Surgical evacuation of the organized pericardial hematoma relieved right heart compression, normalized intracardiac pressures, and resolved the shunt. The pericardium was markedly thickened and adherent to the right ventricle with a fibrotic hematoma, and histopathology revealed fibrotic thickening with inflammatory infiltration and hemosiderin deposition. The patient's respiratory status gradually improved, and she was discharged home without the recurrence of paradoxical cerebral embolism. This case highlights a rare pathophysiological mechanism in which constrictive pericarditis elevates right atrial pressure, unmasking a previously silent PFO and resulting in paradoxical embolism and refractory hypoxemia. Constrictive pericarditis should be considered a potential trigger for right-to-left shunting in patients with unexplained hypoxemia or cryptogenic stroke.

## Introduction

Patent foramen ovale (PFO) is a common congenital interatrial communication present in approximately 15-35% of the adult population [1]. Although often asymptomatic, a PFO can serve as a conduit for paradoxical embolism, particularly when right atrial pressure is elevated, such as in the setting of pulmonary embolism or right heart failure or during the Valsalva maneuver [1,2]. Paradoxical embolism through a PFO can lead to ischemic stroke, systemic embolization, or unexplained hypoxemia [3-6].

PFO results from the incomplete fusion of the septum primum and secundum [7]. Although it typically remains closed in function due to higher left atrial pressure [8], any condition that reverses this gradient can cause right-to-left shunting. The degree of shunting is influenced by defect size and pressure differential [7,9].

Constrictive pericarditis, a chronic inflammatory disorder characterized by the fibrotic thickening of the pericardium and impaired diastolic filling, can lead to elevated right-sided cardiac pressures [10]. Recent expert consensus from the American College of Cardiology has also emphasized early recognition and multimodal imaging for the diagnosis and management of pericardial diseases [11]. Asymmetric pericardial thickening or hematoma formation, especially in postsurgical settings, can selectively compress specific cardiac chambers, most notably the right atrium, further increasing right atrial pressure [12]. The hemodynamic sequelae of constrictive pericarditis include impaired venous return and elevated systemic venous pressure [13]. Right heart compression results in a disproportionate elevation of right atrial pressure while maintaining a relatively normal left atrial pressure, thereby creating the pressure gradient necessary for right-to-left shunting across the PFO [14]. However, the development of paradoxical embolism and hypoxemia due to this mechanism has rarely been reported. Imazio et al. noted that although constrictive pericarditis impairs cardiac filling and increases right-sided pressure, direct evidence of PFO-mediated shunting remains limited [15]. Similarly, Maloku et al. emphasized the diagnostic challenge, as pericardial diseases are infrequently considered precipitating factors for paradoxical embolisms [16].

This case report describes a rare instance of constrictive pericarditis secondary to an organized pericardial hematoma that developed after idiopathic pericarditis, resulting in a right-to-left shunt through a PFO and paradoxical cerebral embolism. To our knowledge, this is the first reported case demonstrating this sequential pathophysiological mechanism.

## Case presentation

A 72-year-old Japanese woman presented with a six-day history of persistent thoracic back pain that worsened upon deep inspiration. At that time, she was afebrile. She visited a local clinic, where the electrocardiogram (ECG) showed ST-segment elevation. The patient was subsequently transported to our hospital by an ambulance.

Her medical history included hypertension and dyslipidemia, for which she was being treated with telmisartan and rosuvastatin. She was a non-smoker and reported drinking approximately 350 mL of beer twice a week.

On admission, her height was 163 cm, her weight was 56.9 kg, and her body mass index was 21.4 kg/m². The patient was alert and well-oriented. Her vital signs were as follows: temperature, 38.5℃; blood pressure, 96/50 mmHg; heart rate, 93 bpm with a regular rhythm; and respiratory rate, 22 breaths/min. Oxygen saturation was 98% on room air. Cardiac murmurs or pulmonary crackles were not observed.

Laboratory findings (Table 1) revealed marked leukocytosis with a white blood cell (WBC) count of 25,800/μL (88% neutrophils), elevated inflammatory markers with a C-reactive protein (CRP) level of 25.7 mg/dL, and a mildly elevated troponin I level (62.9 pg/mL).

**Table 1:** Laboratory test results on admission HPF: high-power field; AST: aspartate aminotransferase; ALT: alanine aminotransferase; LD: lactate dehydrogenase; ALP: alkaline phosphatase; γ-GT: gamma-glutamyl transferase; PT-INR: international normalized ratio of prothrombin time; APTT: activated partial thromboplastin time; n/a: not available

Parameter	Admission	Reference range
Complete blood count		
White blood cell	25.8×10^3^/µL	3.5-9.1×10^3^/µL
Neutrophils	88%	40-74%
Lymphocytes	5%	19-48%
Monocytes	7%	3.4-9%
Eosinophils	0%	0-7%
Red blood cell	343×10^4^/µL	376-500×10^4^/µL
Hemoglobin	10.7 g/dL	11.3-15.2 g/dL
Hematocrit	31.5%	33.4-44.9%
Mean corpuscular volume	91.8 fL	79-100 fL
Platelet	34×10^4^/µL	13-36.9×10^4^/µL
Biochemistry		
Total protein	6.2 g/dL	6.6-8.1 g/dL
Albumin	2.6 g/dL	4.1-5.1 g/dL
Total bilirubin	0.68 mg/dL	0.4-1.5 mg/dL
Direct bilirubin	0.36 mg/dL	0.05-0.23 mg/dL
AST	33 U/L	13-30 U/L
ALT	47 U/L	7-23 U/L
LD	194 U/L	124-222 U/L
Creatine kinase	18 U/L	41-153 U/L
Creatine kinase-MB fraction	1 U/L	0-25 U/L
ALP	219 U/L	106-322 U/L
γ-GT	49 U/L	9-32 U/L
C-reactive protein	25.7 mg/dL	0-0.14 mg/dL
Urea nitrogen	21 mg/dL	8-20 mg/dL
Creatinine	1.03 mg/dL	0.46-0.79 mg/dL
Sodium	141 mmol/L	138-145 mmol/L
Potassium	4.4 mmol/L	3.6-4.8 mmol/L
Chloride	105 mmol/L	100-110 mmol/L
Corrected calcium	9.7 mg/dL	8.4-10.1 mg/dL
Inorganic phosphorus	5.2 mg/dL	2.7-4.6 mg/dL
Magnesium	1.9 mg/dL	1.7-2.5 mg/dL
Plasma glucose	113 mg/dL	70-109 mg/dL
Troponin I	62.9 pg/mL	<6 pg/mL
Brain natriuretic peptide	58.6 pg/mL	0-18.4 pg/mL
Antinuclear antibody	<40 times	<40 times
Coagulation tests		
PT-INR	1.16	0.9-1.2
APTT	38.4 seconds	28.5-40.9 seconds
D-dimer	2.9 µg/mL	0-1 µg/mL
Urinalysis and sediments		
Gravity	1.056	1.005-1.025
pH	5.0	5.0-7.5
Protein	1+	Negative
Glucose	-	Negative
Red blood cells	5-9/HPF	0-2/HPF
White blood cells	1-4/HPF	Negative
Blood culture	Negative	Negative

**Figure 1:**
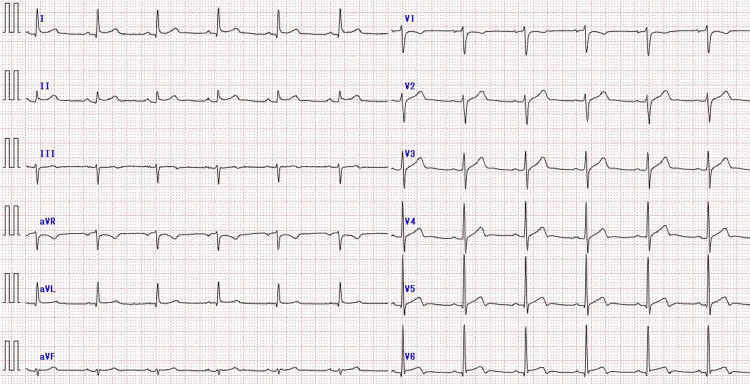
ECG on admission The ECG showing ST-segment elevation in leads I, II, aVL, and V2-V6, without PR-segment depression. ECG: electrocardiogram

The ECG showed ST-segment elevation in leads I, II, aVL, and V2-V6, without PR-segment depression (Figure 1).

**Figure 2:**
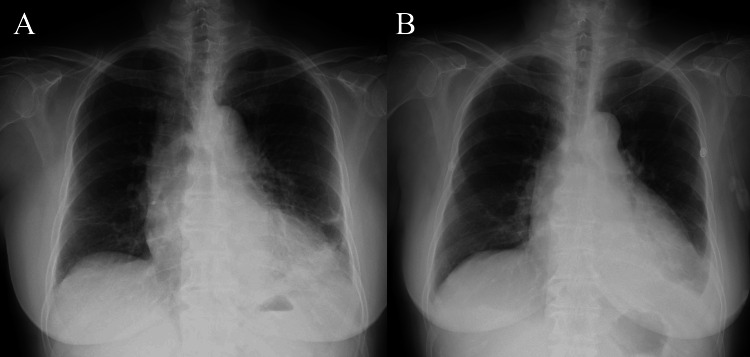
Chest X-rays on admission and on day 17 (A) Chest X-ray on admission showing cardiomegaly and decreased transparency in the bilateral lower lung fields. (B) Chest X-ray on day 17 showing slightly improved transparency in the bilateral lower lung fields compared with admission.

Chest X-ray demonstrated cardiomegaly and decreased transparency in the bilateral lower lung fields (Figure 2A).

**Figure 3:**
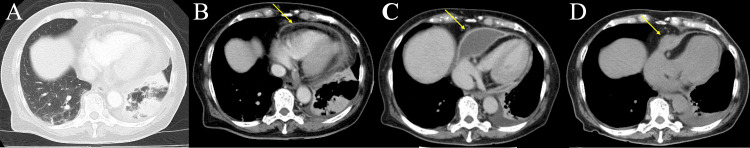
Serial chest CT images demonstrating the evolution of pericardial hematoma (A) Chest CT showing multiple pulmonary nodular infiltrates in the left lung and left pleural effusion. (B) Initial contrast-enhanced CT image on admission showing circumferential pericardial effusion (yellow arrow). (C) Follow-up CT on hospital day 33 demonstrating an encapsulated pericardial collection adjacent to the right atrium (yellow arrow), with an attenuation of approximately 40 HU, suggestive of a hematoma. The dark structure anterior to the right ventricle represents pericardial fat. Left-sided pleural effusion is also present. (D) CT on hospital day 72 demonstrating a persistent encapsulated pericardial collection (yellow arrow) with dense contents adjacent to the right heart, compressing the cardiac chambers and contributing to elevated right atrial pressure. The attenuation measured approximately 50 HU, compatible with a chronic hematoma. The dark structure anterior to the right ventricle represents pericardial fat, and left-sided pleural effusion is also present. CT: computed tomography

Contrast-enhanced computed tomography (CT) subsequently showed localized pericardial effusion compressing the right heart, multiple pulmonary nodular infiltrates in the left lung, and left pleural effusion (Figure 3A-3B).

The following day, coronary angiography revealed no significant stenosis. That same day, she developed a fever of 38°C, and based on the imaging findings, community-acquired pneumonia was diagnosed. Ceftriaxone (2 g every 24 hours) was initiated as empirical therapy. 

**Figure 4:**
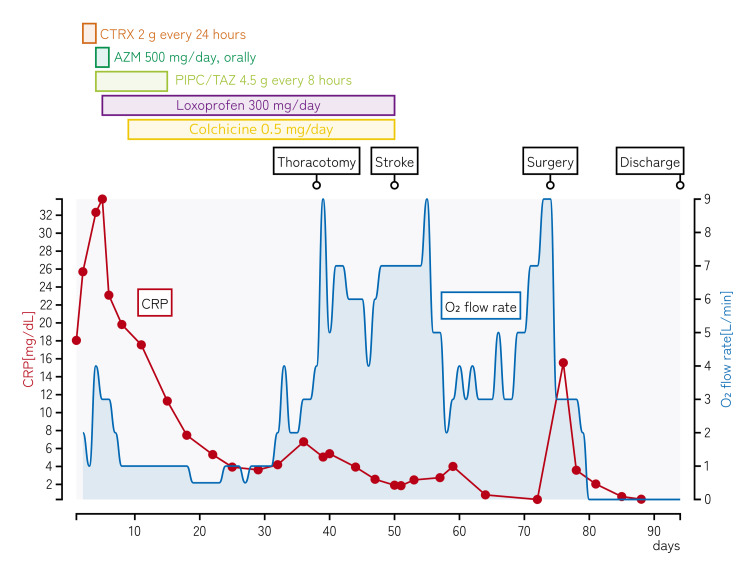
Clinical course of the patient Timeline showing the clinical course during the 94-day hospitalization. The red line with dots represents CRP levels (left y-axis, mg/dL), and the blue line shows oxygen flow rate requirements (right y-axis, L/min). Horizontal bars indicate the duration of antimicrobial and anti-inflammatory therapies. Three major events are marked: thoracotomy (day 39), stroke (day 50), and surgery for pericardial hematoma evacuation (day 74). Note the persistent oxygen requirement despite improvement in CRP levels, which resolved only after surgical intervention. CRP: C-reactive protein; CTRX: ceftriaxone; AZM: azithromycin; PIPC/TAZ: piperacillin/tazobactam; NSAIDs: nonsteroidal anti-inflammatory drugs

By hospital day 4, laboratory tests showed no improvement in inflammation (WBC 25,000/μL and CRP 32.3 mg/dL), and oxygenation had not improved. Therefore, the antibiotic regimen was changed to azithromycin (500 mg/day, orally) and piperacillin/tazobactam (4.5 g every eight hours) to cover atypical pathogens and organisms not susceptible to ceftriaxone. Sputum culture later yielded *Streptococcus pneumoniae* and *Haemophilus influenzae*. Despite receiving 4 L/min supplemental oxygen, her SpO₂ decreased to 94%. Cardiac insufficiency secondary to pericarditis was suspected, and intravenous furosemide was initiated. The clinical course of the patient is shown in Figure 4.

On hospital day 5, loxoprofen (300 mg/day) was initiated for pericarditis-related chest pain, and on day 9, colchicine (0.5 mg/day) was added to treat the presumed idiopathic pericarditis.

By day 17, the levels of inflammatory markers had improved, and chest X-ray showed slightly improved transparency in the bilateral lower lung fields (Figure 2B). Piperacillin/tazobactam was discontinued after two weeks, following the treatment duration typically used for bacteremia. However, despite these improvements, significant hypoxemia persisted, requiring 4 L/min of oxygen. On day 29, follow-up transthoracic echocardiogram (TTE) revealed right-sided dominant pericardial effusion measuring approximately 22-26 mm in thickness anterior to the right ventricle with fibrinous strands, compressing the right heart. The inferior vena cava (IVC) was dilated with poor inspiratory collapse, and the right atrium was not collapsed, suggesting that cardiac tamponade had not yet developed. Contrast-enhanced CT scan on day 33 ruled out pulmonary embolism and deep vein thrombosis but showed that the pericardial effusion had become localized and encapsulated around the right heart (Figure 3C). These conditions were considered as possible causes of right heart pressure elevation, but their exclusion supported constrictive pericarditis as the underlying mechanism. Pericardiocentesis was not performed as no safe access sites were identified on echocardiography. On day 39, a left thoracotomy was performed through the fifth intercostal space to drain the pericardial effusion. The pericardium was incised, releasing a small amount of hemorrhagic effusion with dense adhesions in the cavity. The pericardial space was irrigated using a catheter, but the organized hematoma could not be drained. Pericardial biopsy showed pleural thickening with hemorrhage and inflammation, consistent with chronic pericarditis. The findings were not compatible with tuberculous pericarditis, and immunohistochemistry revealed no malignancy. Pericardial fluid cultures were negative.

**Figure 5:**
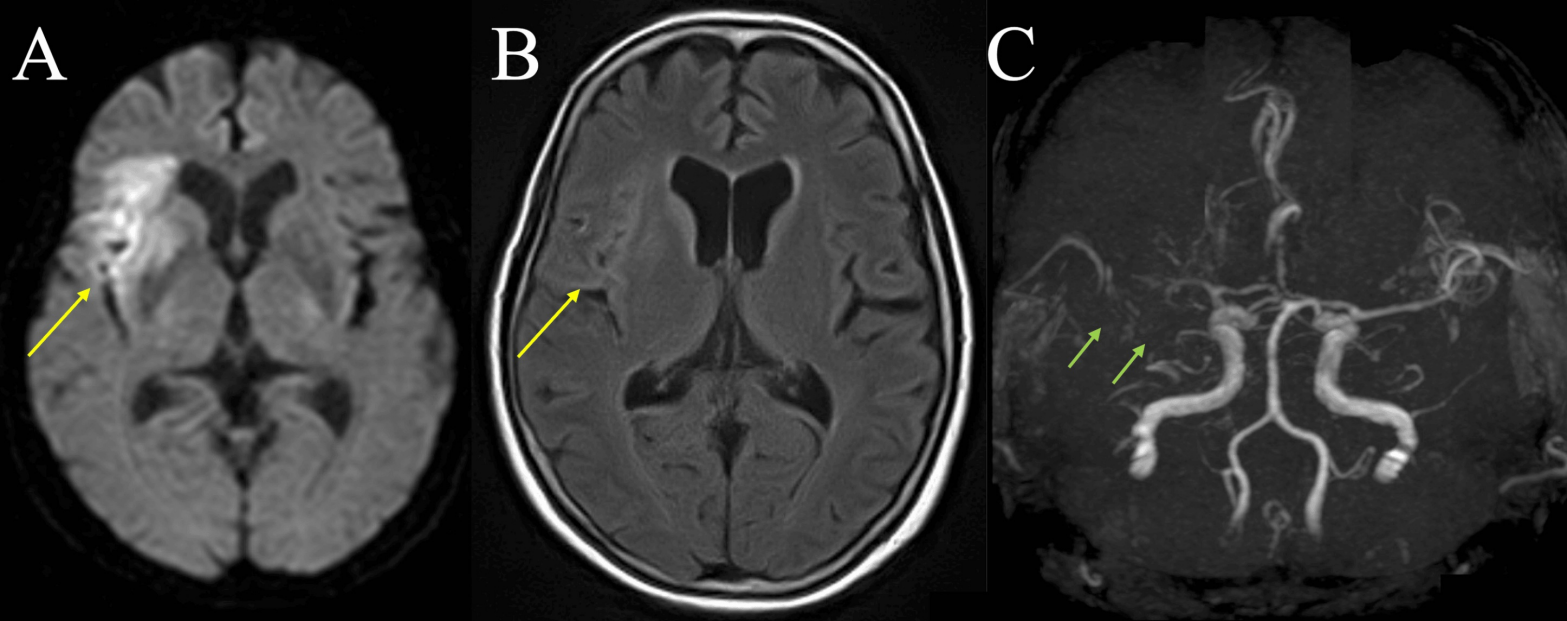
Brain MRI and MRA findings demonstrating multiple embolic strokes (A-C) Brain MRI on hospital day 50. (A) Diffusion-weighted imaging showing a hyper signal intense lesion in the right basal ganglia (yellow arrow). (B) Fluid-attenuated inversion recovery image showing a lesion in the same region. (C) MRA showing the occlusion of the right middle cerebral artery (green arrow). MRI: magnetic resonance imaging; MRA: magnetic resonance angiography

On day 50 of hospitalization, the patient developed acute left-sided weakness. The National Institutes of Health Stroke Scale (NIHSS) score was 6 (facial palsy 2, left arm weakness 1, left leg weakness 2, dysarthria 1), and the modified Rankin Scale (mRS) score was 3. Brain magnetic resonance imaging (MRI) revealed multiple acute infarctions in the right middle cerebral artery (MCA) territory (Figure 5A-5C), consistent with cardioembolic stroke. Owing to the risk of bleeding, anticoagulation therapy was withheld, and only edaravone was administered.

**Figure 6:**
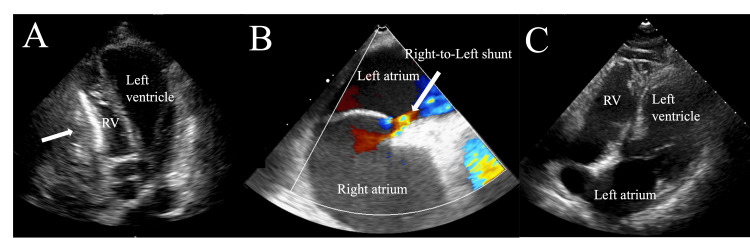
Echocardiographic evidence of right heart compression and intracardiac shunting (A) TTE, apical four-chamber view, showing the compression of the RV due to a pericardial hematoma (white arrow). (B) Intraoperative transesophageal echocardiogram with color Doppler showing right-to-left shunting through the patent foramen ovale with turbulent flow and a mosaic color pattern (white arrow). (C) Follow-up TTE apical four-chamber view on hospital day 82 showing the resolution of right-sided heart compression after the surgical removal of the hematoma. TTE: transthoracic echocardiogram; RV: right ventricle

On day 64, follow-up TTE showed persistent right-sided pericardial effusion and increased echogenicity of the pericardium, which was compressing the right ventricle. The IVC remained dilated with poor inspiratory collapse, and the right atrium was not collapsed, indicating that cardiac tamponade had still not developed (Figure 6A).

The following day, her hemiparesis improved. The NIHSS score was 2 (facial palsy 1, dysarthria 1), and the mRS score was 1. Follow-up MRI showed decreased signal intensity on diffusion-weighted imaging with improved flow in the right MCA.

Given the persistent hypoxemia despite the resolution of systemic inflammation, presence of right heart compression on echocardiography, and occurrence of embolic strokes with no identifiable source, a right-to-left shunt was suspected. Although right heart catheterization was considered, it was anticipated that advancing the catheter into the pulmonary artery would be unsuccessful due to the significant compression of the right heart chambers. Because this compression was already evident on imaging and surgical intervention was considered inevitable, the decision was made to proceed directly with the surgery. On hospital day 68, a lung ventilation-perfusion scan showed no significant mismatch, effectively ruling out pulmonary embolism. Follow-up CT on day 72 confirmed a persistent hematoma that had become organized and caused significant compression of the right heart (Figure 3D).

**Figure 7:**
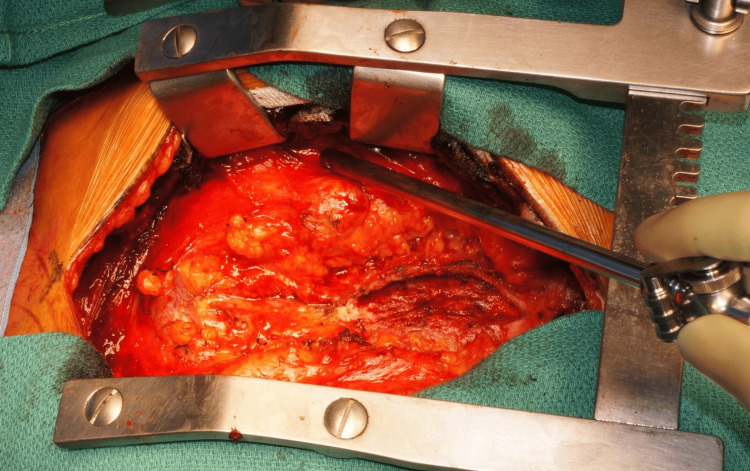
Intraoperative photograph Intraoperative photograph showing the operative field after the removal of the organized pericardial hematoma.

On hospital day 74, the patient underwent pericardiotomy under general anesthesia. Intraoperative transesophageal echocardiography (TEE) confirmed a right-to-left shunt through the PFO (Figure 6B). The surgery was performed via a median sternotomy. The pericardium was markedly thickened and adherent to the right ventricle, and a fibrotic thrombus was firmly attached to the epicardial surface. The hematoma was completely removed except for the area near the inferior vena cava, where strong adhesion made further dissection hazardous due to the risk of venous injury. After decompression, right heart filling improved, although the right-to-left shunt through the PFO persisted immediately after surgery. The pericardium was left open, and the chest was closed in the usual manner (Figure 7).

**Figure 8:**
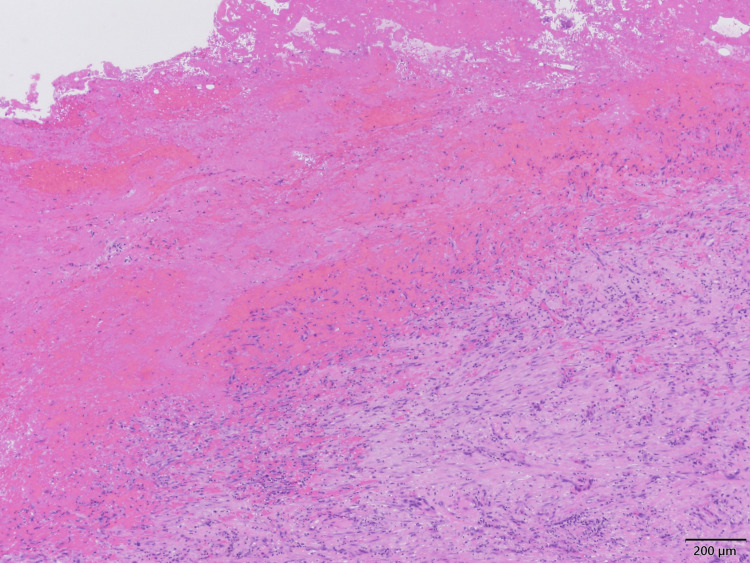
Histopathological findings of the pericardium Histopathological findings of the pericardium showing fibrotic thickening with inflammatory cell infiltration and an attached hematoma exhibiting organization and hemosiderin deposition (H&E stain, ×40). H&E: hematoxylin and eosin

Histopathological examination revealed fibrotically thickened pericardial tissue with lymphocytic, plasma cell, and foamy cell infiltration. An attached hematoma showed organization and hemosiderin deposition, indicating a subacute to chronic process (Figure 8).

On day 82, follow-up TTE confirmed the resolution of the right heart compression and showed no evidence of a right-to-left shunt (Figure 6C).

On hospital day 88, right heart catheterization showed pressures in the right atrium (mean 6 mmHg), right ventricle (end-diastolic pressure 9 mmHg), and pulmonary artery (mean 12 mmHg), with a pulmonary capillary wedge pressure of 5 mmHg. The right atrial and pulmonary capillary wedge pressures were nearly equal, no oxygen step-ups were observed, and there was no evidence of pulmonary hypertension or residual right-to-left shunt.

The patient was discharged home on hospital day 94. Her respiratory condition remained stable during follow-up, and no further paradoxical cerebral embolism occurred.

## Discussion

**Figure 9:**
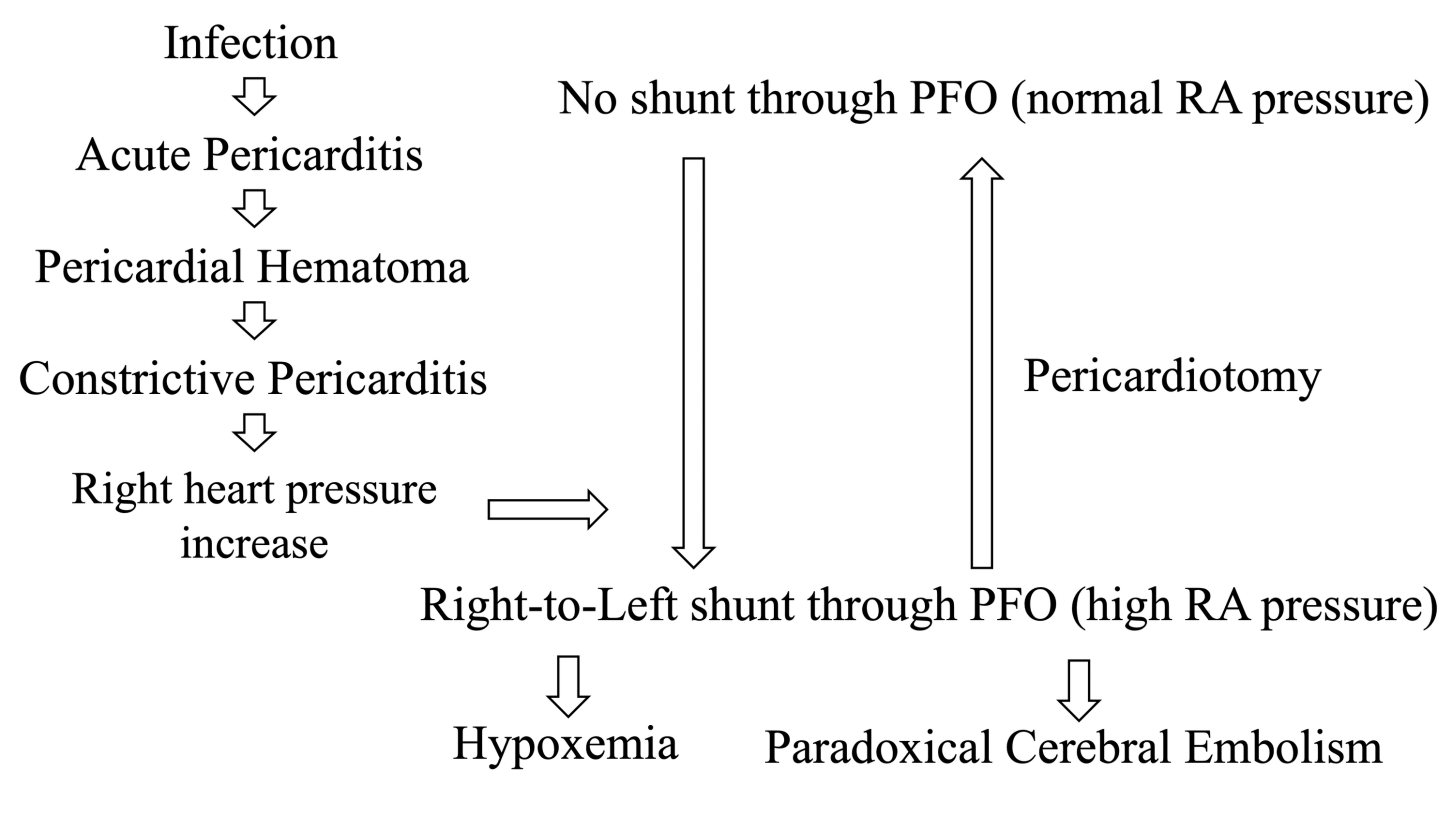
Proposed mechanism of right-to-left shunting through a PFO induced by constrictive pericarditis The schematic illustrates how fibrotic transformation of the pericardial hematoma led to compression of the right heart, resulting in elevated RA pressure. When left atrial pressure remained normal, the altered interatrial pressure gradient enabled right-to-left shunting across a previously silent PFO. This mechanism may result in paradoxical embolism, causing cryptogenic stroke, and allow venous blood to bypass the pulmonary circulation, leading to systemic hypoxemia. RA: right atrial; PFO: patent foramen ovale

This case illustrates a rare but clinically important scenario in which constrictive pericarditis leads to a right-to-left intracardiac shunt via a previously asymptomatic PFO, resulting in both paradoxical cerebral embolism and hypoxemia. The proposed mechanism, illustrated in Figure 9, likely involves elevated right atrial pressure due to impaired right ventricular filling caused by pericardial fibrosis. This elevated pressure gradient facilitates right-to-left shunting through the PFO [2,10].

Previous reports have linked acute pericarditis to hemorrhagic pericardial effusion and, when organization occurs, to constrictive physiology [15]. The risk is increased in patients receiving oral anticoagulants or with renal failure/uremic pericarditis [17]. Inflammation-related angiogenesis may also cause bleeding even without antithrombotic exposure. In our patient, although antithrombotic therapy was withheld, an organized hematoma developed and compressed the right heart, unmasking a previously silent PFO and resulting in right-to-left shunting, consistent with prior case reports [18]. These findings underscore the importance of cautious anticoagulant use and close echocardiographic follow-up in patients with pericarditis-associated effusion. 

Typically, TEE with a bubble study is the gold standard for demonstrating a right-to-left shunt via the PFO [8]. This procedure was not performed preoperatively in our patient because the clinical evidence was highly suggestive of an intracardiac shunt. This evidence included findings of cardioembolic stroke, hypoxemia, and significant right heart compression on imaging [5]. Given the marked pericardial thickening and compression of the right ventricle, we suspected that constrictive pericarditis involving the right heart was contributing to the patient's hemodynamic compromise. Therefore, we decided to proceed with surgical intervention without additional invasive testing. Ultimately, a definitive diagnosis was established intraoperatively with TEE, which enabled the real-time imaging of the right-to-left shunt through the PFO [9]. 

Right-to-left shunting through a PFO typically occurs during transient elevations in right atrial pressure, such as those seen in pulmonary embolism or chronic obstructive pulmonary disease or during the Valsalva maneuver [5,16]. In contrast, chronic right heart compression, as seen in constrictive pericarditis, is a less common but plausible cause, as demonstrated in our case [16].

Paradoxical embolism is a potentially serious complication of PFO, especially in the presence of prothrombotic conditions [3,4]. In our patient, the delayed recognition of a PFO-mediated shunt underscores the importance of considering intracardiac shunting in cases of cryptogenic stroke when no other embolic source has been identified [5,6]. The absence of atrial fibrillation, intracardiac thrombi, and carotid artery disease further supported the diagnosis of paradoxical embolism via a PFO, which was confirmed by TEE, demonstrating a right-to-left shunt. Notably, postsurgical right heart catheterization revealed normalized intracardiac pressure and no residual shunting, confirming that the shunt was dynamic and reversible. These findings support the concept that pericardial compression can transiently elevate right atrial pressure and unmask previously silent PFO [10,16].

This case emphasizes the need to consider PFO-related right-to-left shunting in patients with unexplained hypoxemia or embolic stroke, particularly those with pericardial disease. Timely recognition and appropriate intervention, such as surgical decompression in cases similar to ours, may prevent recurrent embolic events and improve outcomes.

## Conclusions

This case demonstrates that localized constrictive pericarditis can serve as a direct trigger for paradoxical embolism via a PFO. Proactive evaluation of intracardiac shunts is recommended for patients with unexplained hypoxemia or cryptogenic stroke, particularly when pericardial disease is suspected. Timely diagnosis and surgical intervention are essential for preventing recurrence and improving patient outcomes.

## References

[1] Koutroulou I, Tsivgoulis G, Tsalikakis D, Karacostas D, Grigoriadis N, Karapanayiotides T (2020). Epidemiology of patent foramen ovale in general population and in stroke patients: a narrative review. Front Neurol.

[2] Teshome MK, Najib K, Nwagbara CC, Akinseye OA, Ibebuogu UN (2020). Patent foramen ovale: a comprehensive review. Curr Probl Cardiol.

[3] Kent DM, Saver JL, Ruthazer R (2020). Risk of paradoxical embolism (RoPE)-estimated attributable fraction correlates with the benefit of patent foramen ovale closure: an analysis of 3 trials. Stroke.

[4] Ntaios G, Papavasileiou V, Sagris D, Makaritsis K, Vemmos K, Steiner T, Michel P (2018). Closure of patent foramen ovale versus medical therapy in patients with cryptogenic stroke or transient ischemic attack: updated systematic review and meta-analysis. Stroke.

[5] Kent DM, Ruthazer R, Weimar C (2013). An index to identify stroke-related vs incidental patent foramen ovale in cryptogenic stroke. Neurology.

[6] Kuramoto J, Kawamura A, Dembo T, Kimura T, Fukuda K, Okada Y (2015). Prevalence of patent foramen ovale in the Japanese population - autopsy study. Circ J.

[7] Zisa D, Faletra FF, Wessler BS, Halin NJ, Reddy P, Patel AR, Pandian NG (2020). Ridges and pouches: a case series of anomalous atrial septal fusion. CASE (Phila).

[8] Kutty S, Sengupta PP, Khandheria BK (2012). Patent foramen ovale: the known and the to be known. J Am Coll Cardiol.

[9] Gomperts N, Fowler R, Horlick E, McLaughlin P (2008). A broken heart: right-to-left shunt in the setting of normal cardiac pressures. Can J Cardiol.

[10] Restelli D, Carerj ML, Bella GD (2023). Constrictive pericarditis: an update on noninvasive multimodal diagnosis. J Cardiovasc Echogr.

[11] Wang TK, Klein AL, Cremer PC (2025). 2025 Concise clinical guidance: an ACC expert consensus statement on the diagnosis and management of pericarditis: a report of the American College of Cardiology Solution Set Oversight Committee. J Am Coll Cardiol.

[12] Luong C, Kim JM, Wong GC, Klein R, Brunner N (2021). Loculated pericardial effusion: an uncommon cause of left ventricular outflow tract obstruction. JACC Case Rep.

[13] Sengupta PP, Eleid MF, Khandheria BK (2008). Constrictive pericarditis. Circ J.

[14] Konecny T, Khanna AD, Novak J (2014). Interatrial pressure gradients during simulated obstructive sleep apnea: a catheter-based study. Catheter Cardiovasc Interv.

[15] Imazio M, Gaita F, LeWinter M (2015). Evaluation and treatment of pericarditis: a systematic review. JAMA.

[16] Maloku A, Hamadanchi A, Günther A, Aftanski P, Schulze PC, Möbius-Winkler S (2024). Patent foramen ovale (PFO): history, diagnosis, and management. Rev Cardiovasc Med.

[17] Rhabneh L, Rout P (2025). Uremic pericarditis. StatPearls [Internet].

[18] Schuiteman E, Verrill T, Mina N, Dalal B (2017). Constrictive pericarditis-induced shunting through a PFO: persistence despite pericardiectomy. Respir Med Case Rep.

